# Strain-Induced Domain Structure and Its Impact on Magnetic and Transport Properties of Gd_0.6_Ca_0.4_MnO_3_ Thin Films

**DOI:** 10.1021/acsomega.1c04904

**Published:** 2021-12-09

**Authors:** Azar Beiranvand, Elmeri Rivasto, Hannu Huhtinen, Petriina Paturi

**Affiliations:** Wihuri Physical Laboratory, Department of Physics and Astronomy, University of Turku, FI-20014 Turku, Finland

## Abstract

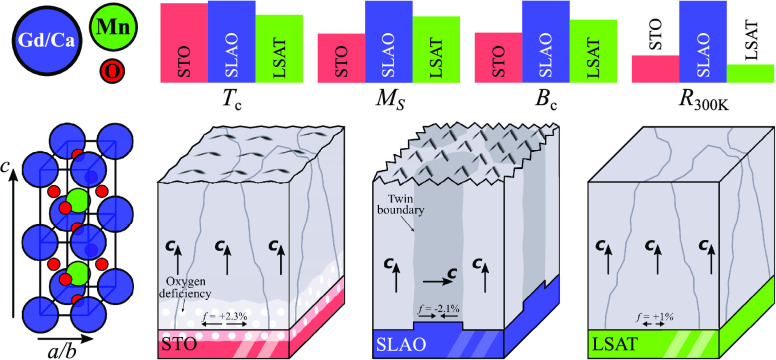

The evolution of lattice strain on crystallographic domain structures and magnetic properties of epitaxial low-bandwidth manganite Gd_0.6_Ca_0.4_MnO_3_ (GCMO) films have been studied with films on different substrates: SrTiO_3_, (LaAlO_3_)_0.3_(Sr_2_AlTaO_6_)_0.7_, SrLaAlO_3_, and MgO. The X-ray diffraction data reveals that all of the films, except the films on MgO, are epitaxial and have an orthorhombic structure. Cross-sectional transmission electron microscopy (TEM) shows lattice mismatch-dependent microstructural defects. Large-enough tensile strain can increase oxygen vacancies concentration near the interface and can induce vacancies in the substrate. In addition, a second phase was observed in the films with tensile strain. However, compressive strain causes dislocations in the interface and a mosaic domain structure. On the other hand, the magnetic properties of the films, including saturation magnetization, coercive field, and transport property depend systematically on the substrate-induced strain. Based on these results, the choice of appropriate substrate is an important key to obtaining high-quality GCMO film, which can affect the functionality of potential device applications.

## Introduction

The mixed-valence A_1–*x*_B_*x*_MnO_3_ perovskite manganites, where A and B are rare-earth and divalent alkaline elements, have recently become the focus of extensive research due to their unusual magnetic and magnetoresistance properties.^1−4^ Among the perovskites, the low-bandwidth manganites, such as Pr_1–*x*_Ca_*x*_MnO_3_ (PCMO) and Gd_1–*x*_Ca_*x*_MnO_3_ (GCMO), are particularly interesting due to the stable charge ordering (CO) state in the whole doping range, leading to several important features like resistive switching and spin memory effect.^5−8^ Moreover, the Gd-based low-bandwidth perovskites not only show the CO state near room temperature but also exhibit specific magnetic features, particularly the reversal magnetization at low temperature and in the applied magnetic field, leading to a ferrimagnetic ground state.^9−12^ The structural and physical properties of such materials are strongly dependent on the deposition technique and the lattice mismatch with the substrate, which results in uniaxial strain.^13−15^ The strain can be responsible for phase separation^16,17^ and modification of the relation between the lattice parameters in the direction perpendicular to growth (*c*) and the one in the parallel plane (*a*).^18^ On the other hand, the fabrication of high-quality perovskite thin films by controlling the strain and the lattice mismatch between the bulk and substrate can affect the electrical and magnetic properties of manganites.^10,19^

In our previous paper,^20^ positron annihilation studies of Gd_0.6_Ca_0.4_MnO_3_ (GCMO) thin films grown on SrTiO_3_ (STO) showed that most of the oxygen vacancies and open volume defects are in the interface region between the film and the substrate. This is due to the transfer of oxygen from STO to the film bulk to compensate oxygen vacancies in the GCMO lattice. Hence, other substrates could be a solution to solve this problem. In this work, the high-quality Gd_0.6_Ca_0.4_MnO_3_ thin films have been deposited on different substrates, and their microstructural, magnetic, and electrical properties have been investigated. These results show the effect of lattice mismatch and pave the way for integrating GCMO films on silicon, which is the ultimate goal.

## Results and Discussion

The epitaxial GCMO films were grown on SrTiO_3_ (STO), (LaAlO_3_)_0.3_(Sr_2_AlTaO_6_)_0.7_ (LSAT), SrLaAlO_3_ (SLAO), and MgO substrates by pulsed laser deposition (PLD). As expected, the GCMO films grow diagonally on the substrates. The lattice mismatch between the GCMO bulk and diagonal of all substrate is determined by the formula *f* = (√2*a*_S_ – *a*_B_)/*a*_B_, where *a*_B_ = 5.424 Å is the average of GCMO bulk values in the in-plane direction.^21^ The lattice parameters of the substrates and the lattice mismatches in the in-plane direction are presented in Table 1.

**Table 1:** Lattice Parameters of the Substrates and Lattice Mismatches between the Average of the Lattice Parameters of GCMO Bulk in the In-Plane Direction and along the Diagonal of the Substrate’s Unit Cell

substrate	*a*_s_ (Å)	*c*_s_ (Å)	*f* (%)
STO	3.905		+2.350
LSAT	3.868		+0.84
SLAO	3.756	12.636	–2.088
MGO	4.213		+9.847

### Magnetic Phases and Transitions

**Figure 1:**
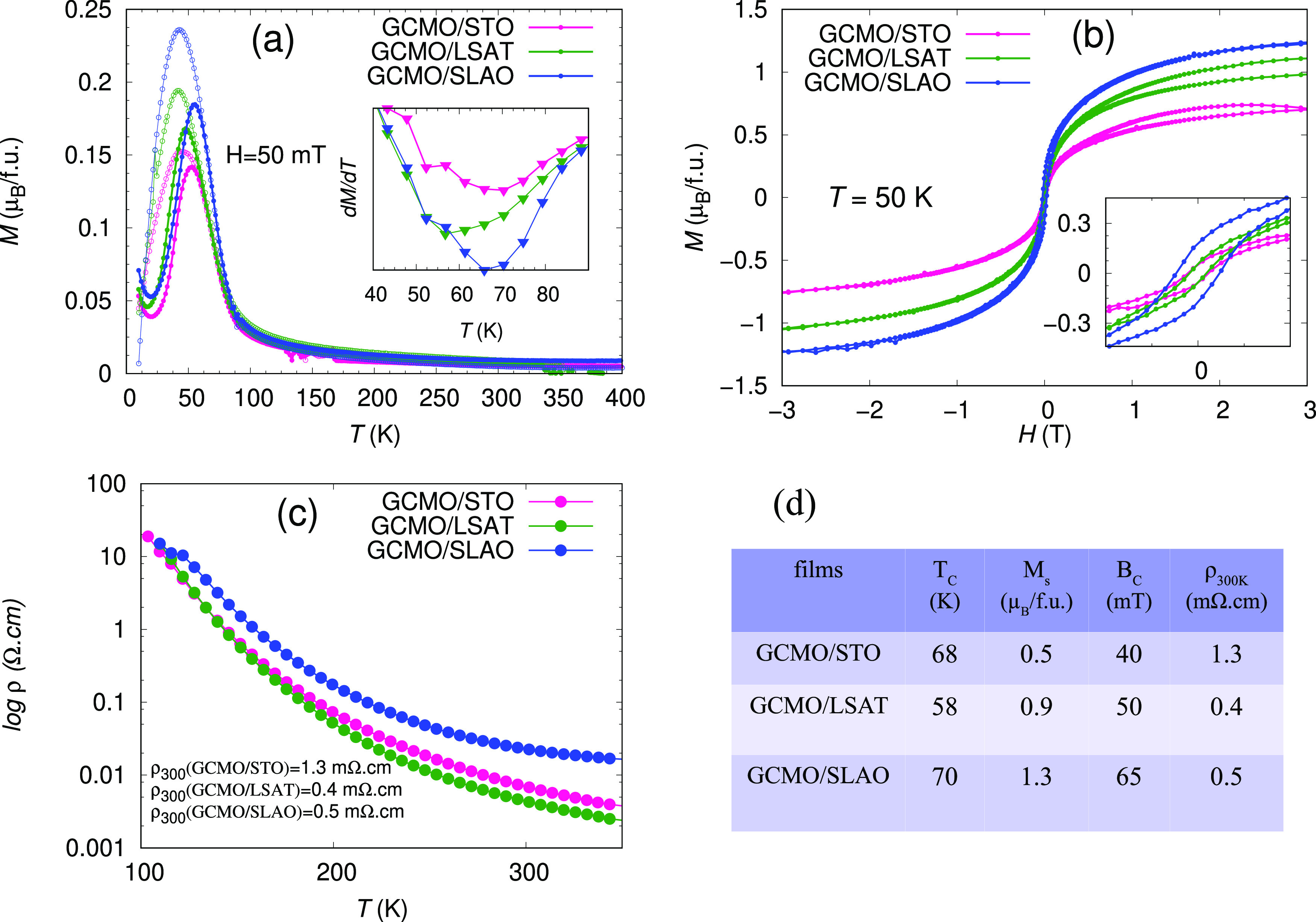
(a) Temperature dependences of magnetizations measured in a 50 mT magnetic field for GCMO/STO, GCMO/LSAT, and GCMO/SLAO films. ZFC curves are shown with filled symbols and FC curves with open symbols. The inset is the first derivative of the FC curves. (b) Hysteresis loops of GCMO films grown on different substrates and measured at 50 K. The insets show a closer view in the range of the zero field. (c) shows the *R*(*T*) curves of the GCMO films. The data below 100 K is inaccessible due to high resistivity. (d) Most important magnetic and electric properties of the films: *T*_C_ is the Curie temperature, *M*_s_ (5 T) is the magnetization at 50 K and 5 T, *B*_C_ is the coercive field at 50 K, and ρ_300K_ is the resistivity at room temperature.

The typical zero-field-cooled (ZFC) and field-cooled (FC) temperature dependence of magnetizations are measured with an external magnetic field of 50 mT applied parallel to the film surface (Figure 1a). As reported in the previous literature,^9,11,19^ at low temperature, the net magnetization of GCMO components is due to the Gd spins and the Mn spins, which are oriented antiparallel with each other. As temperature increases, the magnetic moments of the Mn ions dominate, reaching a maximum at around 50 K. The strength of the maximum depends on the Mn^4+^/Mn^3+^ ratio, lattice distortion, and oxygen content in the perovskite lattice.^13,14,23^ In our case, the maximum for the GCMO/STO film has lower values compared to that for the GCMO/LSAT and the GCMO/SLAO films. Previously,^20^ we showed that GCMO/STO films contain oxygen vacancies in the lattice and GCMO takes oxygen from STO to compensate the vacancies. It seems that oxygen vacancies and other defects in this film can suppress the double exchange (DE) interaction, resulting in lower ferromagnetic alignment. The existence of defects and microstructure properties for all of the films will be discussed by TEM measurements in the following section.

Upon further heating, the magnetization decreases rapidly, changing the sample to the paramagnetic state. The ordering temperature, *T*_C_, to paramagnetic state is determined from the first derivative of FC measurement, d*M*/d*T*. The *T*_C_ is approximately 68 K for STO, 58 K for LSAT, and 70 K for SLAO. The compressive strain increases *T*_C_ and tensile strain decreases *T*_C_ as follows^24^
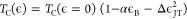
1where ϵ_B_ = (2ϵ_100_ – ϵ_001_) is the bulk strain,  is the Jahn-Teller (JT) strain, α = (1/*T*_C_)(d*T*_C_/dϵ_B_), and Δ = (1/*T*_C_)(d^2^*T*_C_/dϵ_JT_^2^). The magnitudes of α and Δ represent the relative weights for the symmetry-conserving bulk and the symmetry-breaking JT strains, respectively. The second term in eq 1 is related to the change of the kinetic energy of the carriers with strain and the third term is related to the electron localization due to the splitting of the e_g_ level caused by the static JT distortion. According to eq 1, the compressive strain with negative ϵ_B_ increases *T*_C_. One could expect that the GCMO/SLAO and GCMO/LSAT films have higher *T*_C_ when compared with that of the GCMO/STO film with tensile strain (positive ϵ_B_). However, the GCMO/LSAT has the lowest *T*_C_ among the three films, and *T*_C_ for the GCMO/SLAO film is close to that of the GCMO/STO film. A possible explanation for this discrepancy is that the unit cell distortion is larger in the GCMO/SLAO and GCMO/LSAT films when compared with that in the GCMO/STO film. It seems that the effect of lattice distortion becomes dominant over the strain effect in these films, leading to decreased *T*_C_.

Figure 1b shows *M*(*H*) curves for all of the films measured at 50 K. The diamagnetic signal from the substrate and the sample holder measured at 400 K has been subtracted from all the curves. All of the films show a hysteresis loop with a negligible coercive field at 10 K (not shown), and no saturation in the magnetization curves can be obtained up to 5 T, which confirms the ferrimagnetic background of the GCMO at low temperature.^25^ GCMO/SLAO shows the highest magnetization at 5 T (*M*_s_) among all of the films. This is in agreement with the *M*(*T*) measurement. At 50 K, the magnetic properties are mainly due to the magnetic moments of Mn ions. The magnetization increases linearly with the applied magnetic field and it does not saturate up to 5 T for any of the films. This can be associated with the appearance of the ferromagnetic state due to Mn^3+^–Mn^4+^ interaction within the AFM matrix due to Mn^3+^–Mn^3+^ and Mn^4+^–Mn^4+^ interactions in these films. The coercive fields at 50 K are 40, 50, and 65 mT for GCMO/STO, GCMO/LSAT, and GCMO/SLAO, respectively. This implies that more domain wall pinning sites exist in the GCMO/SLAO film when compared with those in GCMO/STO and GCMO/LSAT films.

The temperature dependence of resistivity measurements (*R*(*T*)) shows the semiconducting behavior for all of the samples, which means that the resistivity increases gradually as temperature decreases (see Figure 1c). One can see that the GCMO/SLAO film exhibits the highest resistivity in the temperature range of 10–400 K among the three films. The higher resistivity in the GCMO/SLAO film can be due to the greater number of defects caused by a large compressive lattice mismatch between the film and the substrate, as will be discussed later in the paper. However, the resistivity values for the GCMO/LSAT film with the smallest film–substrate mismatch are close to those for the GCMO/STO film with a larger tensile mismatch. This can be attributed to the increased number of other defects in the GCMO/LSAT film when compared with the GCMO/STO film.

### Structural and Microstructural Properties

**Figure 2:**
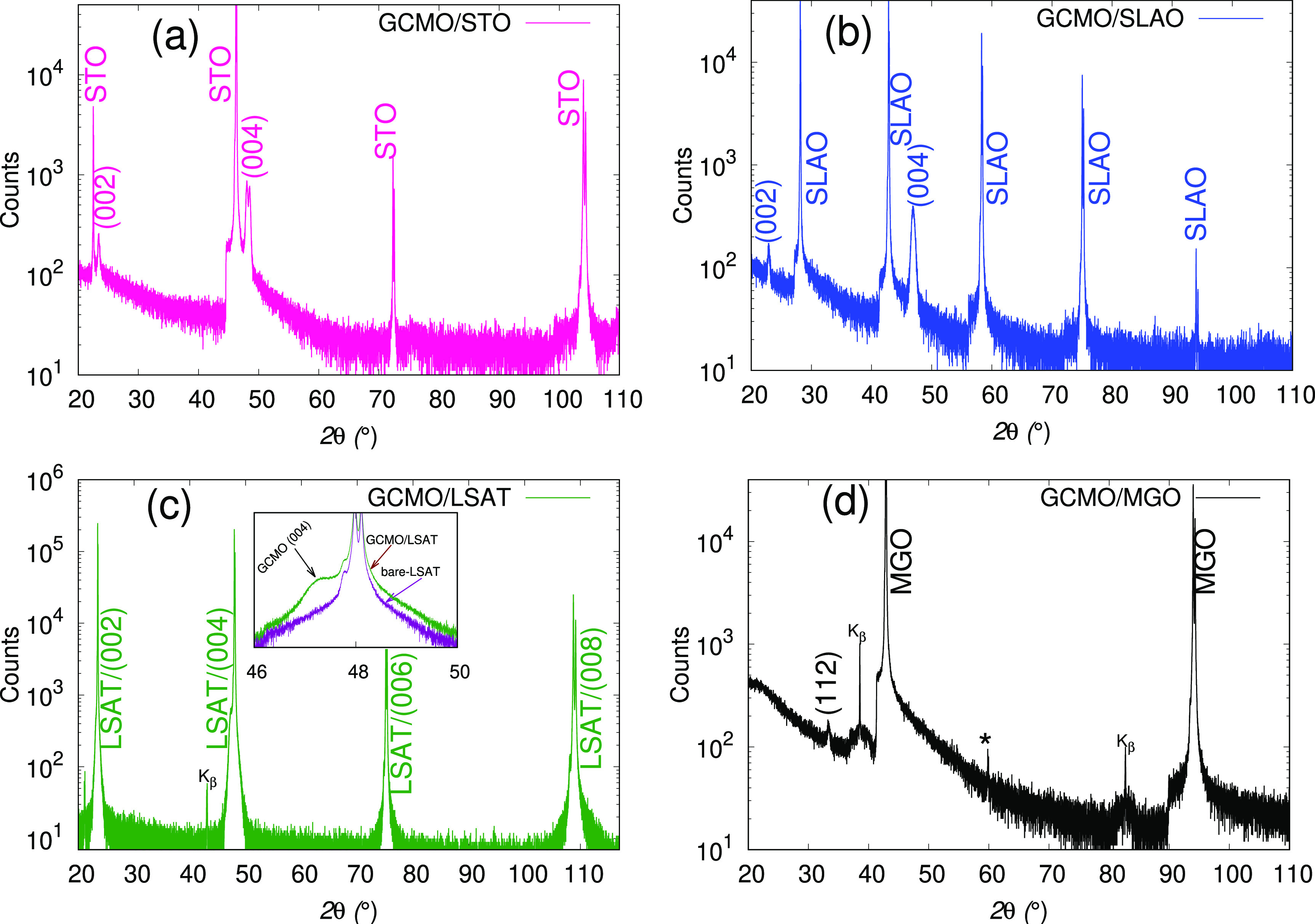
Room-temperature θ–2θ scans measured in the (00*l*) direction for GCMO thin films grown on (a) STO, (b) SLAO, (c) LSAT, and (d) MGO substrates. The intensity is given in the logarithmic scale. The peak marked with an asterisk (*) is an unidentified peak or arises from the sample holder. The inset shows the detail of the (004) peak together with the bare substrate.

The XRD θ–2θ scans of the GCMO films grown on STO, LSAT, SLAO, and MgO substrates (hereafter GCMO/STO, GCMO/LSAT, GCMO/SLAO, and GCMO/MgO) in the (00*l*) direction are shown in Figure 2. In GCMO/STO, GCMO/LSAT, and GCMO/SLAO films no diffraction peaks from secondary phases or other orientations are observed. This indicates that the films are single-phase, epitaxially grown, and completely textured. Due to the large mismatch between the GCMO film and the MgO substrate, only the (112) peak was observed in the XRD pattern, which indicates that this film is not textured but polycrystalline, and thus the structure of this film is not studied further in this work.

The relatively narrow peaks in the (004) direction indicate that the *c* lattice parameters in the GCMO/STO and GCMO/SLAO films are very uniform along the film thickness. On the other hand, the (224) peak width in the ϕ-direction of the GCMO/STO film is slightly broader than that of the GCMO/SLAO film (Table 2), indicating a greater number of low angle grain boundaries in the GCMO/STO film. For the GCMO/LSAT films, determining the peak widths in 2θ or ϕ directions is not possible due to the overlap of the peaks because of the minimal lattice mismatch between the substrate and the film.

**Table 2:** Lattice Parameters, the Substrate-Induced Strain ε*_a_* and ε*_c_* along the In-Plane and Out-of-Plane Directions, Respectively, the Peak Widths from 2θ (004) Peak and ϕ (224) Peak as well as the Thickness (*d*) of the GCMO Films on Different Substrates Calculated from the Room-Temperature XRD and XRR Data^a^

substrate	*a* (Å)	*b* (Å)	*c* (Å)	ε*_a_* (%)	ε*_c_* (%)
STO	5.41(5)	5.46(3)	7.52(3)	0.2	0.03
SLAO	5.30(4)	5.30(2)	7.72(3)	–2.22	2.7
LSAT	5.34(1)	5.37(5)	7.61(4)	–1.08	1.2

	Δ2θ (deg)	Δϕ (deg)	*r*_a_ (nm)	*d* (nm)
STO	0.68	2.7	0.69	56
SLAO	0.52	1.7	0.9	45
LSAT			0.34	52

a The RMS roughness (*r*_a_) is calculated from the images taken by atomic force microscopy. The numbers in the brackets correspond to the standard deviations of the least significant digits of the parameter values. The peaks of GCMO on LSAT overlap with the substrate peaks and therefore the peak widths cannot be determined.

The lattice parameters of the films were determined from the XRD data to calculate the change of unit cell volume, Δ*V*, and substrate-induced strain by ε*_a_* = (*a*_F,*a*_ – *a*_B_)/*a*_B_ in the in-plane and out-of-plane (ε*_c_*) directions. The values are listed in Table 2. From θ–2θ and two-dimensional ϕ-scans of (022) and (224) peaks, the GCMO/LSAT film peaks are at the same positions as the substrate peaks in the in-plane direction and therefore we consider the substrate peaks as the film peaks to determine the lattice parameters (inset of Figure 2c). The XRD θ–2θ scans of all of the films measured in the ⟨112⟩ and ⟨224⟩ directions are shown in the Supporting Information.

The in-plane strain, ε*_a_* is tensile for the GCMO/STO film and is compressive for the GCMO/SLAO film, as expected from the lattice mismatch. For the GCMO/LSAT film, the lattice mismatch is negligible in the in-plane direction (see Table 1); however, the film is under compressive strain, about 1%, in this direction. This indicates that the lattice mismatch is not the only factor that affects the film lattice parameters. Oxygen vacancy concentration, Mn^4+^/Mn^3+^ ratio, and other defects also have an important role in the final unit cell volume.^26,27^

The out-of-plane strain, ε_*c*_ is tensile for both GCMO/SLAO and GCMO/LSAT films, in accordance with the in-plane compressive strain in these films (see Table 2). It means that ε_*c*_ has an opposite sign to the in-plane strain and Poisson’s ratio is positive for these films. For the GCMO/STO film, the out-of-plane lattice parameter is expanded with a small magnitude strain although the in-plane strain is tensile. The increase in lattice parameters in both in-plane and out-of-plane directions has been observed also for other manganites films grown on STO.^28^ It has also been observed, that the length of the *c-*axis can be affected by the deposition parameters and structural defects in the films prepared by the pulsed laser deposition method.^29,30^ Hence, the substrate-induced strain cannot alone explain the differences observed in the *c* lattice parameter.

The surface microstructures of the films grown on different substrates are very similar in all of the films (not shown here), as measured by atomic force microscopy. The surface RMS roughness values, given in Table 2, show that all of the films are extremely smooth, indicating almost two-dimensional growth with strong film–substrate bonding, low film surface energy, and high substrate surface energy.^31^ However, the GCMO/LSAT film is clearly the smoothest, having the RMS roughness value only half of the value of the other films. This is probably due to the small lattice misfit and strain energy, which allows the film to grow in the layer-by-layer mode rather than by an island growth mechanism.^32^ The thickness of the films shown in Table 2 is determined by XRR measurements. From the data, one can see that with the same number of laser pulses during the deposition process, the film on SLAO is the thinnest one. Previous literature showed that the growth rate of manganite thin films depends on the orientation and surface instabilities of the substrate.^33^ This suggests that, in addition to the strain effect, other properties of the substrate surface like the adhesion or the termination could affect the growth of initial layers, i.e., first few unit cells and therefore the growth mode of the film.^34,35^

**Figure 3:**
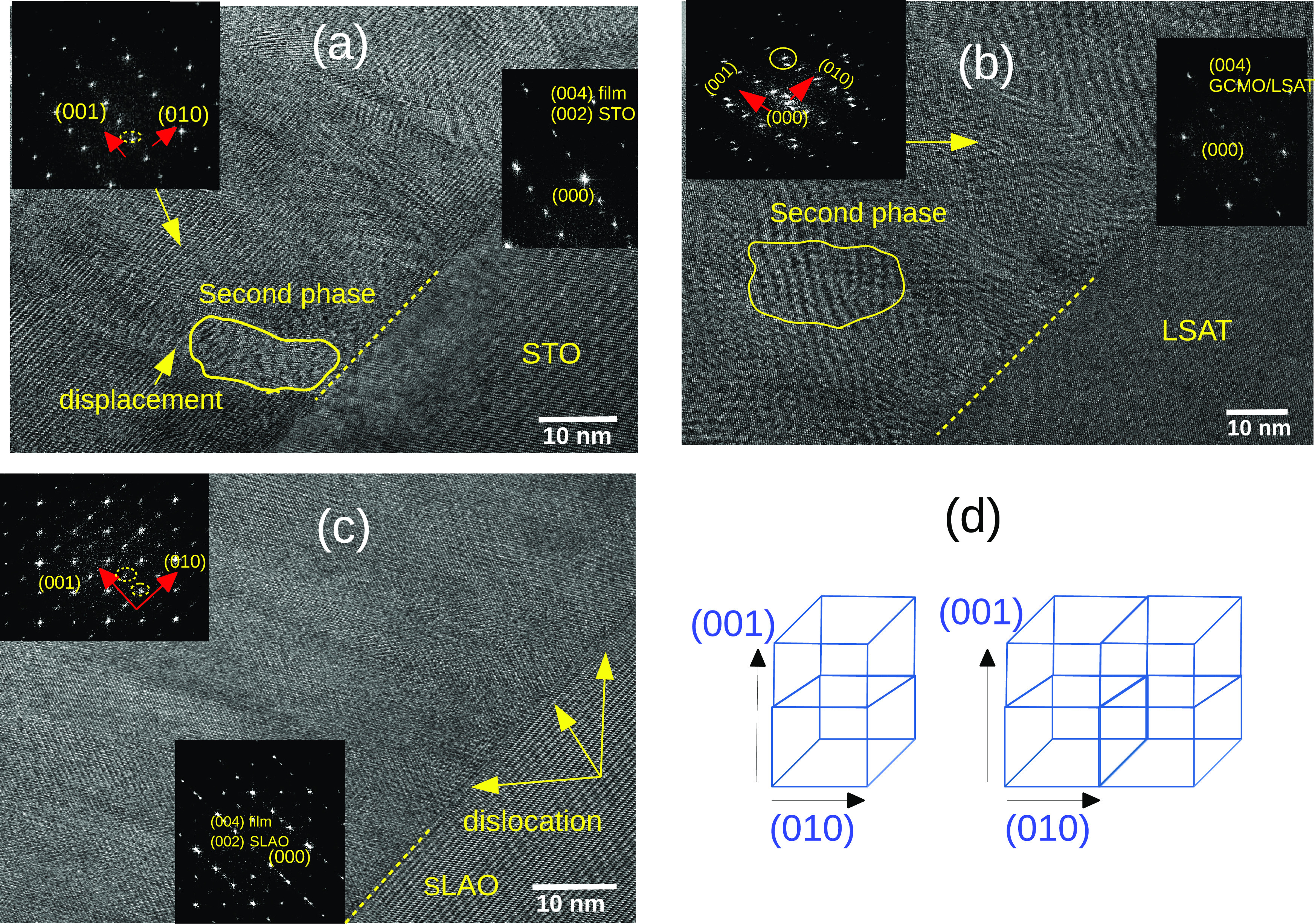
Cross-sectional HRTEM images along the ⟨100⟩ direction of GCMO on (a) STO, (b) LSAT, and (c) SLAO substrates. The insets are FFTs of the film (upper right corner) and diffraction patterns of the film–substrate interface (upper left corner). Superstructure reflections for half-integer peaks in the FFTs are dash-circled. The *a*/*b*-axes variation in the GCMO/LSAT film are shown by a circle in the FFT image. (d) Sketch shows the unit cell doubling along the (001) direction (left) and along both the (001) and (010) directions (right).

Figure 3 shows the cross-sectional HRTEM images of all films. As can be seen, the film/substrate interface (shown by dashed line) is sharp, without any contractable characteristics of interfacial layers for all of the films. Cell doubling of the perovskite cubic unit cell was confirmed by a fast Fourier transform (FFT) of the images in the interface region, which show half-integer reflection of (0 1/2 0) and (0 0 1/2). On the STO substrate, the FFT image shows only the (0 0 1/2) reflection. We can say that the doubling of the unit cell is in the (001) direction, which means that the superlattice reflections are along the direction perpendicular to the film/substrate interface, as also schematically illustrated in Figure 3d. In addition, the nanocluster of a secondary phase, probably oxygen-deficient GCMO, has been recognized in the GCMO/STO film. The density of these nanoclusters is higher near the interface than on the top part of the film. The formation of the second phase can be due to oxygen vacancies in this system. In our previous report,^20^ positron annihilation spectroscopy measurements showed that the GCMO can take oxygen from the STO substrate during the deposition to compensate for the oxygen deficiency in the lattice, and thus the defect concentration could be larger near the interface region. The existence of the second phase, which affects the Mn^4+^/Mn^3+^ ratio and the number of oxygen vacancies could suppress the DE interaction, decreasing magnetization. This is in agreement with the magnetization measurements.

On LSAT, the FFT image shows that the GCMO unit cells are oriented along the *c*-axis, but there is slight variation in the in-plane orientation of the *a*/*b*-axes (see the inset of Figure 3b). Thus, it seems that, due to the small mismatch between film and the substrate, the strain from the substrate is not strong enough to control the unit cell orientation during the growing process. Other factors such as oxygen vacancy or impurity concentration can order the GCMO unit cells. The impurity nanoclusters, which are spread through the film, were observed in the GCMO/LSAT film.

The GCMO/SLAO film has a sharp interface with the substrate and shows clear dislocations, which are indicated by arrows in Figure 3c and in the TEM image in the Supporting Information. The misfit dislocations can be due to the large compressive lattice mismatch between the film and the substrate in the in-plane direction (Figure 3c). The large compressive substrate–film lattice mismatch can lead to the formation of the dislocation to the relief of the strain, as described by the domain matching epitaxy (DME) model.^37^ Those are rather common in other perovskites under compressive strain films.^36^ Some of these dislocations result in columnar defects. The film exhibits superlattice reflections in both directions perpendicular and parallel to the interface between the film and substrate (see the inset of Figure 3c,d) meaning that the cell doubling happened along both the ⟨010⟩ and ⟨001⟩ directions. The FFT image shows both (0 1/2 0) and (0 0 1/2) reflections, which indicate a multidomain microstructure of the film.

**Figure 4:**
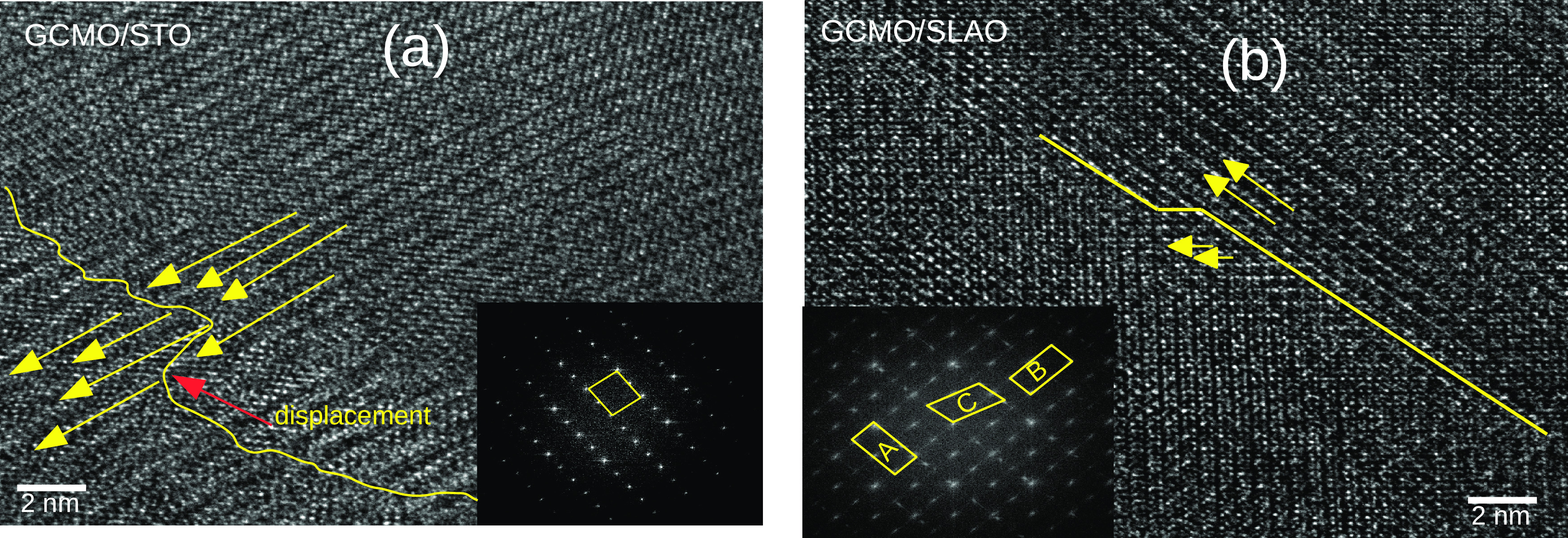
Planar-view HRTEM image of the GCMO heterostructure on (a) STO and (b) SLAO substrates. The solid lines show the domain walls and the yellow arrows exhibit the domain orientation. The red arrow shows the displacement in the domain wall. The insets are the FFT images of the GCMO films and the parallelogram show the unit cell orientation.

**Figure 5:**
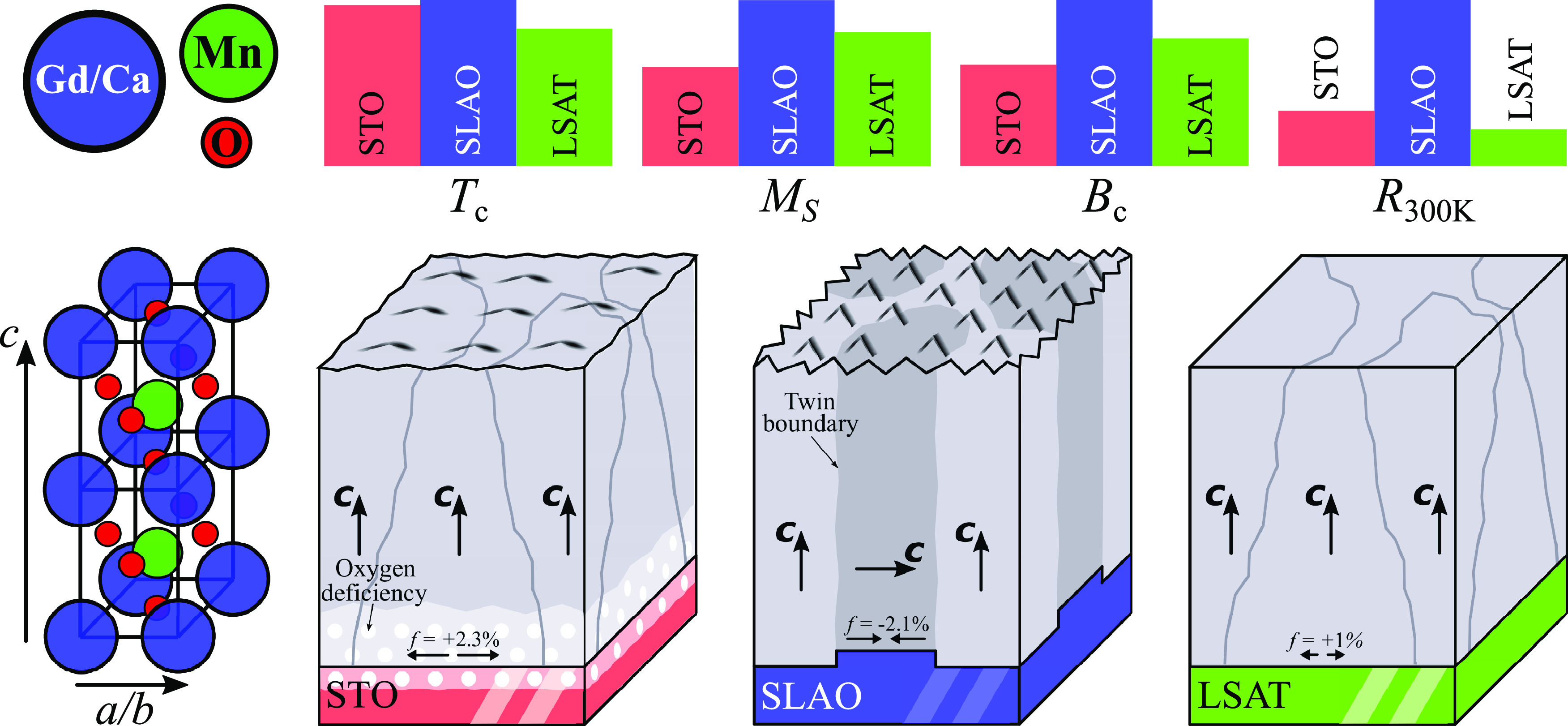
Domain orientations shown schematically for GCMO films grown on different substrates. The GCMO/STO and GCMO/LSAT films are *c*-oriented in the out-of-plane direction. The GCMO/SLAO has twinned domains with *c*-axis oriented in the out-of-plane and in-plane directions. The GCMO/STO has oxygen deficiency in the interface region, which is shown by white dots. The *f* is the lattice mismatch between GCMO bulk and the substrates. The bars show variations in Curie temperature (*T*_C_), magnetization at 5 T (*M*_s_), and resistivity at room temperature (*R*_300_) in the films.

To investigate the domain orientation, a close view of HRTEM images was explored (Figure 4). In the GCMO/STO film, domains are mostly oriented perpendicular to the interface and just a small displacement can be seen in the domain walls (see Figure 4a). This can be confirmed by the FFT image, which shows the reflection of the *c*-oriented unit cell of the GCMO only in the (001) direction (the inset of Figure 4a). Figure 4b shows two domains perpendicular and inclined with respect to the interface, which are shown by arrows for the GCMO/SLAO film. The FFT image of the local region shows various diffraction patterns in this film (see the inset of Figure 4b). The two twinned domains (here labeled as A and B) exhibit *c*-axis along the (001) and (010) directions. When these domains coalesce at a later growth stage, they form a twin boundary. Across the twin boundary, the third domain (labeled C) with the *cb*-plane tilted 8° clockwise from the (010) direction was observed. This can be attributed to the mosaic domain microstructure, resulting in dislocations, which are observed in the interface between the film and the substrate. The crystallography of the domain orientations is schematically illustrated in Figure 5. The existence of the twin boundaries and domains with different orientations in the GCMO/SLAO film explains the observed greater coercive field and resistivity. The twin boundaries can act as pinning sites for the domain walls, which consequently increases the coercive field.^38^ In addition, the multiple orientations of the crystalline domains can decrease electron hopping by increasing the O–Mn bond length and decreasing the bond angle, which leads to increased resistivity. However, the domains in GCMO/STO and GCMO/LSAT are *c*-oriented and thus oxygen vacancies in the GCMO lattice are more pronounced.

## Conclusions

Our findings indicate that the lattice mismatch and strain can affect the GCMO films structurally, magnetically, and electronically. The GCMO film on the MGO substrate with a large lattice mismatch does not grow epitaxially. On SLAO, the domains are orientated differently, but epitaxially. This leads to an increase in *B*_c_ and resistivity, which can be explained by the formation of pinning domain walls and decreased electron hopping through the film. Overall, the structure of the film is very clean and uniform and thus *M*_s_ and *T*_C_ are higher. On STO and LSAT, the films contain a secondary-phase nanocluster not observable in XRD, which can affect the *M*_s_ and *T*_C_. Both these films are *c-*oriented; however, the structure on LSAT is more distorted due to negligible lattice mismatch. These results give limits for the possible lattice mismatches when integrating GCMO film into semiconductor structures for future device applications.

## Experimental Methods

The epitaxial GCMO films were grown by pulsed laser deposition (PLD) with 2000 pulses of XeCl-laser (λ = 308 nm) with the energy density of 1.3 J/cm^2^ and frequency of 5 Hz was used for the depositions of all samples. The deposition temperature was 700 °C, and the films were kept at the atmospheric pressure of oxygen for 10 min at 700 °C, before cooling them down to room temperature, after the deposition. The details of GCMO targets synthesized via the solid-state method are described in ref (12). The structural properties of the thin films were explored using a Philips X’pert PRO diffractometer with a Schulz goniometer and a PixCel 1D detector. The θ–2θ scans over (00*l*), (0*kk*), and (*hh*2*h*) peaks were done to determine the lattice parameters with the Nelson–Riley method.^22^ In addition, a two-dimensional ϕ-scan of the (224) peak was measured to determine the in-plane mosaic spread of the films. The substrate-induced strain between the measured lattice parameters of the thin films and the polycrystalline bulk is calculated as ε_*a*_ = (*a*_F,*a*_ – *a*_B_)/*a*_B_ in the in-plane and out-of-plane directions. The thickness of the films was measured using X-ray reflectivity (XRR) measurements with Philips X’pert PRO equipped with an X-ray mirror and a proportional counter detector. High-resolution TEM (HRTEM) imaging was performed with a JEOL JEM-2200FS electron microscope combined with a 200 kV field emission gun (FEG) and in an in-column energy filter (Omega Filter). Also, a probe-corrected scanning TEM using high-angle annular dark-field imaging (HAADF STEM) was performed with TITAN 80-300 at the voltage of 200 kV. The TEM images were also used to determine the film thickness.

The temperature dependence of the zero-field-cooled (ZFC) and field-cooled (FC) magnetization was measured between temperatures of 10 and 400 K with a Quantum Design SQUID magnetometer in a 50 mT external magnetic field. The magnetic hysteresis curves were recorded in magnetic fields up to 5 T at temperatures of 10, 50, 100, and 400 K. The external field *B* was always oriented along the GCMO (110) axis in the planes of the films. The resistivity measurements were done in a standard four-probe method with the constant current of 0.5 μA in the temperature range from 10 to 400 K with the Physical Property Measurement System (PPMS, Quantum Design).

## Supporting Information Available

θ–2θ scans of the (022) and (224) peaks for the GCMO film grown in STO, LSAT, and SLAO substrates. HRTEM image of the GCMO/SLAO film (ZIP)
