# Supplementary material for: Strain-Induced Domain Structure and Its Impact on Magnetic and Transport Properties of Gd0.6Ca0.4MnO3 Thin Films
Source: ACS Omega. 2021 Dec 9;6(50):34572–9. doi: 10.1021/acsomega.1c04904 (PMC8697384; doi:10.1021/acsomega.1c04904)
Supplement: Supplementary file 1 — ao1c04904_si_001.zip [file ao1c04904_si_001.zip › supplementary file/supplementary file.pdf]

Supporting information for: Strain induced domain structure and its impact on magnetic and transport properties of $Gd_{0.6}Ca_{0.4}MnO_3$ thin films

Azar Beiranvand,* Elmeri Rivasto, Hannu Huhtinen, and Petriina Paturi

Wihuri Physical Laboratory, Department of Physics and Astronomy, University of Turku, FI-20014 Turku, Finland

E-mail: azabei@utu.fi

The XRD measurements

The $\theta - 2\theta$ scans of (022) and (224) peaks for GCMO film grown in STO, LSAT and SLAO substrates are shown in Figure S1.

Microstructure properties

In order to show the periodic formation of dislocations in interface between film and substrate, the HRTEM image of GCMO/SLAO film is displayed in Figure S2 .

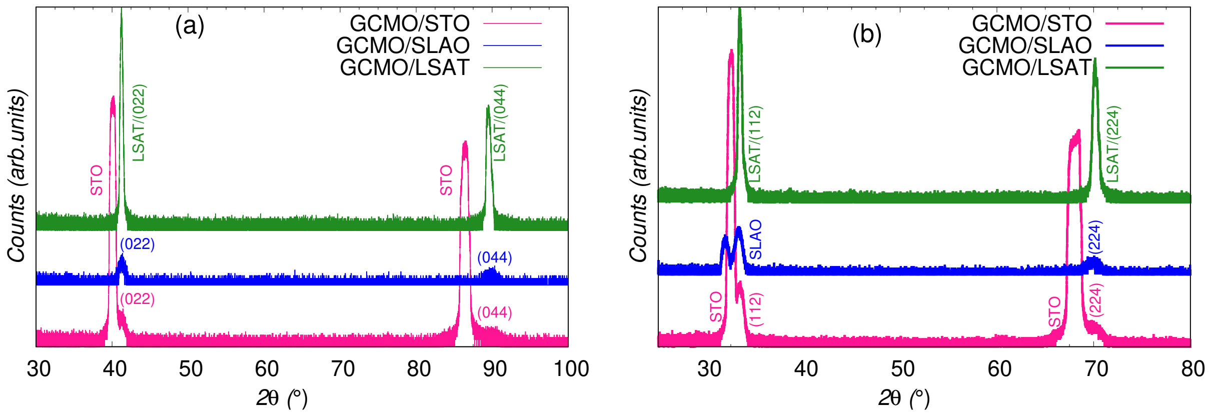

Figure S1: The room temperature $\theta - 2\theta$ scans measured in (a) $\langle 112 \rangle$ and (b) $\langle 224 \rangle$ directions for GCMO thin films grown on different substrates.

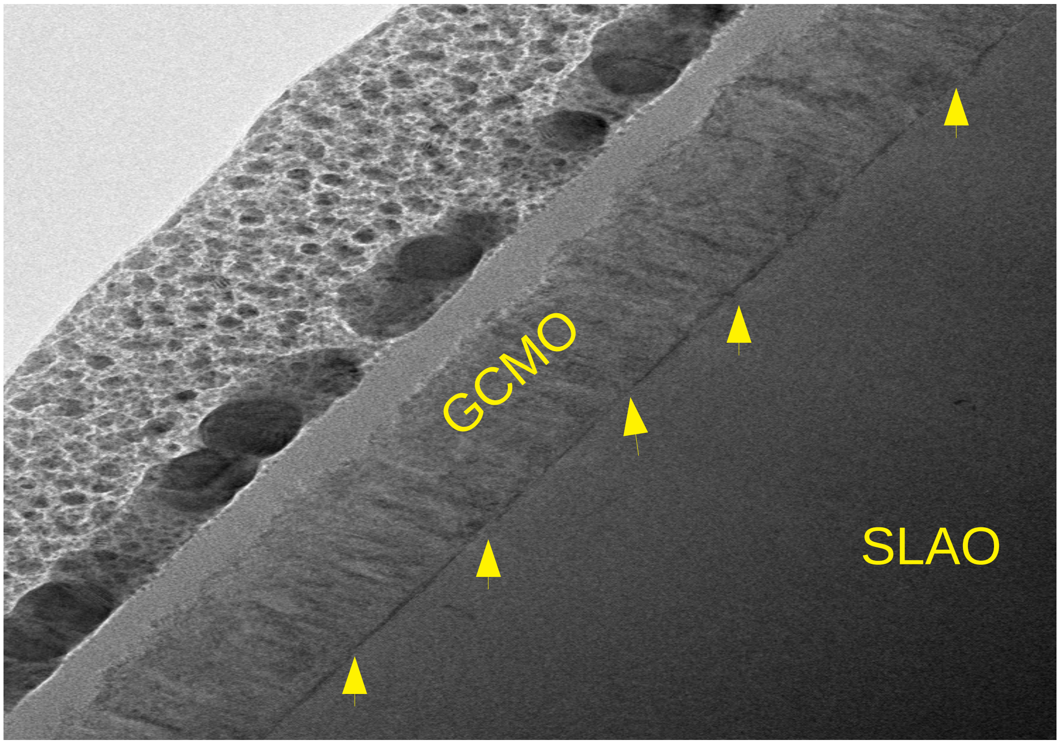

Figure S2: The HRTEM image of GCMO/SLAO film to show the overall view of the interface region with the periodic dislocations shown by the arrows.
