# Supplementary figures and images for: Strain-Induced Domain Structure and Its Impact on Magnetic and Transport Properties of Gd0.6Ca0.4MnO3 Thin Films
Source: ACS Omega. 2021 Dec 9;6(50):34572–9. doi: 10.1021/acsomega.1c04904 (PMC8697384; doi:10.1021/acsomega.1c04904)

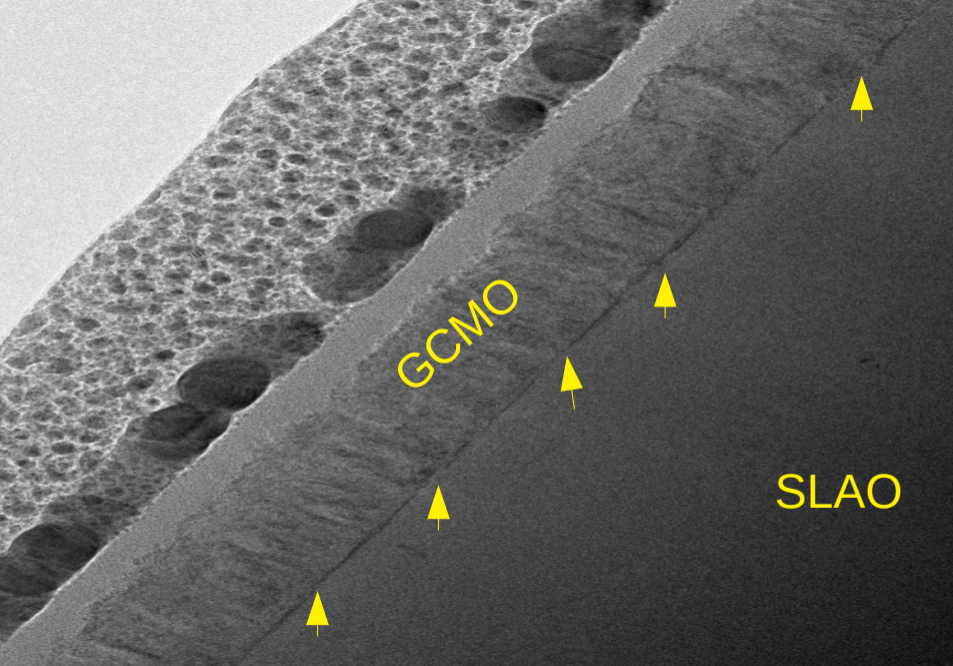

Supplement: Supplementary file 1 — ao1c04904_si_001.zip [file ao1c04904_si_001.zip › supplementary file/SLAO-TEM.pdf]

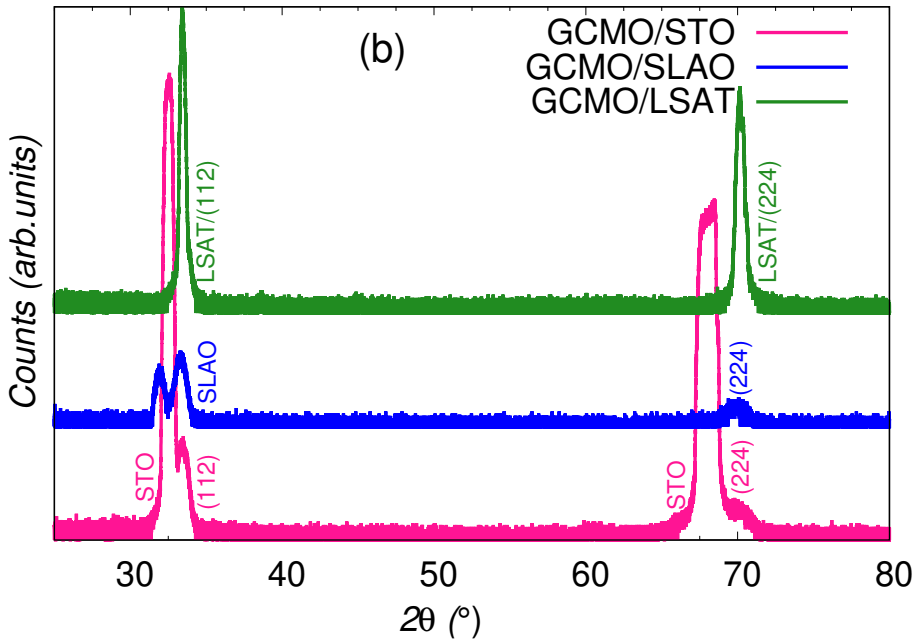

Supplement: Supplementary file 1 — ao1c04904_si_001.zip [file ao1c04904_si_001.zip › supplementary file/112-xrd.pdf]

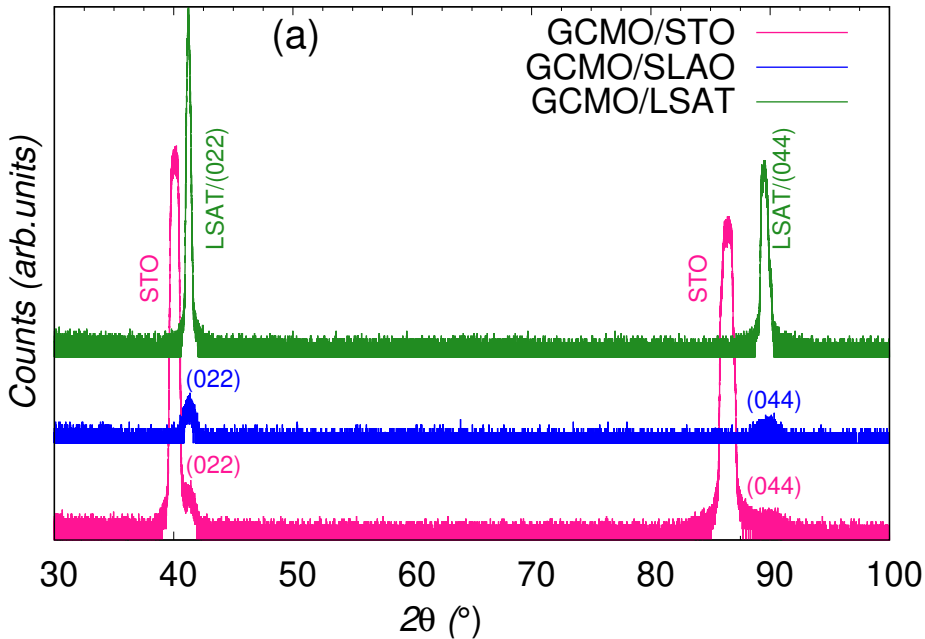

Supplement: Supplementary file 1 — ao1c04904_si_001.zip [file ao1c04904_si_001.zip › supplementary file/011-xrd.pdf]
